# Supplementary figures and images for: Galvanic current activates the NLRP3 inflammasome to promote Type I collagen production in tendon
Source: eLife. 2022 Feb 24;11:e73675. doi: 10.7554/eLife.73675 (PMC8896827; doi:10.7554/eLife.73675)

Figure 2-source data 1

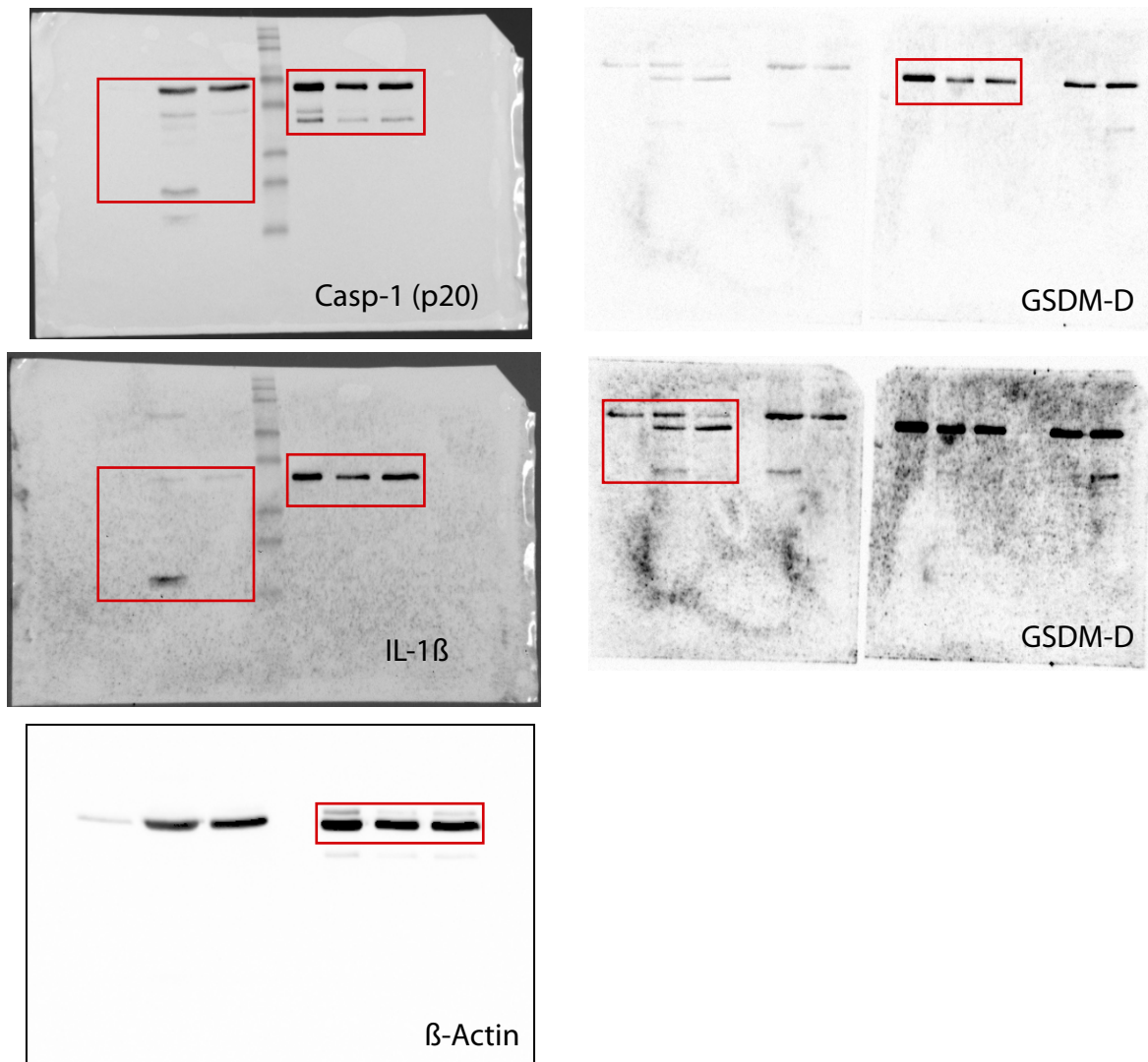

Supplement: Figure 2—source data 1. [file elife-73675-fig2-data1.pdf]

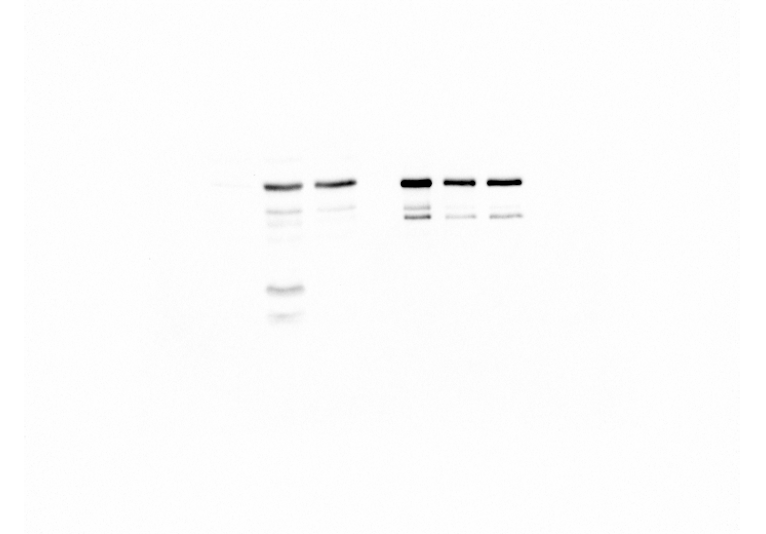

Supplement: Figure 2—source data 2. [file elife-73675-fig2-data2.zip › Figure 2-source data 2/WB_Casp1.tif]

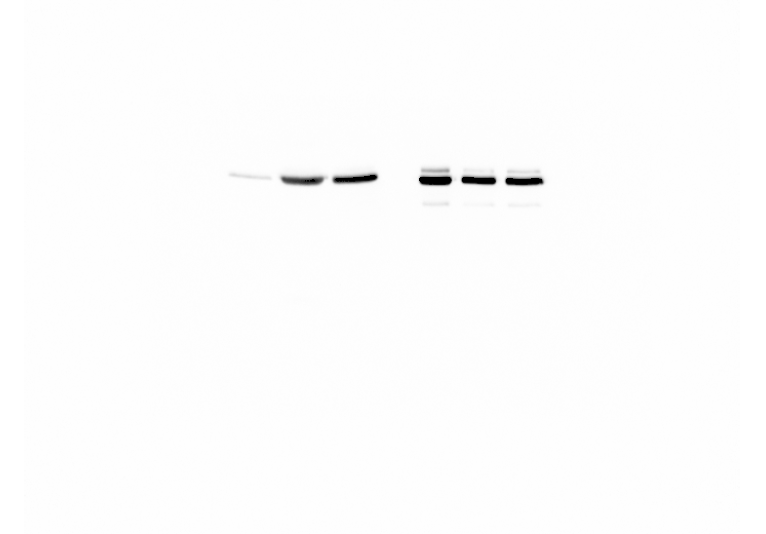

Supplement: Figure 2—source data 2. [file elife-73675-fig2-data2.zip › Figure 2-source data 2/WB-bActin.tif]

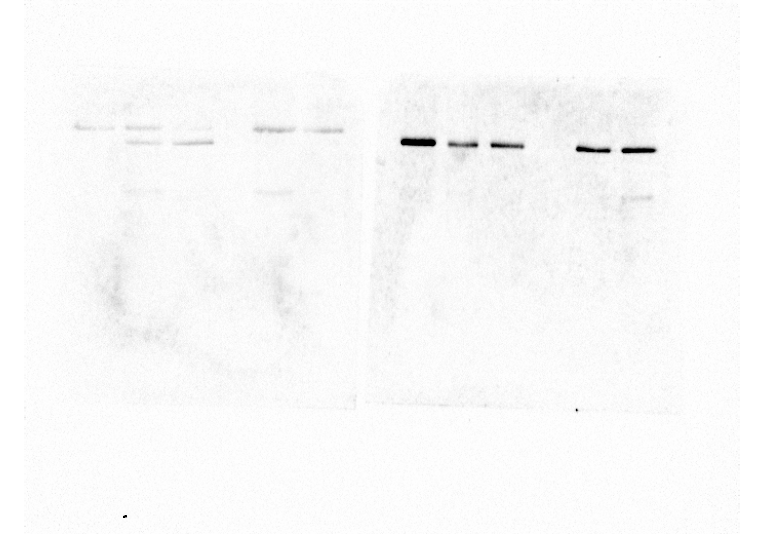

Supplement: Figure 2—source data 2. [file elife-73675-fig2-data2.zip › Figure 2-source data 2/WB_GSDMD-1.tif]

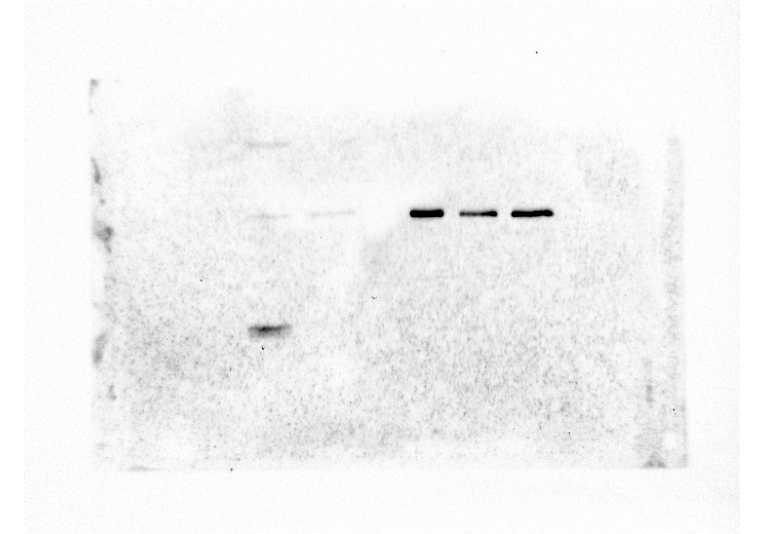

Supplement: Figure 2—source data 2. [file elife-73675-fig2-data2.zip › Figure 2-source data 2/WB_IL1B.tif]

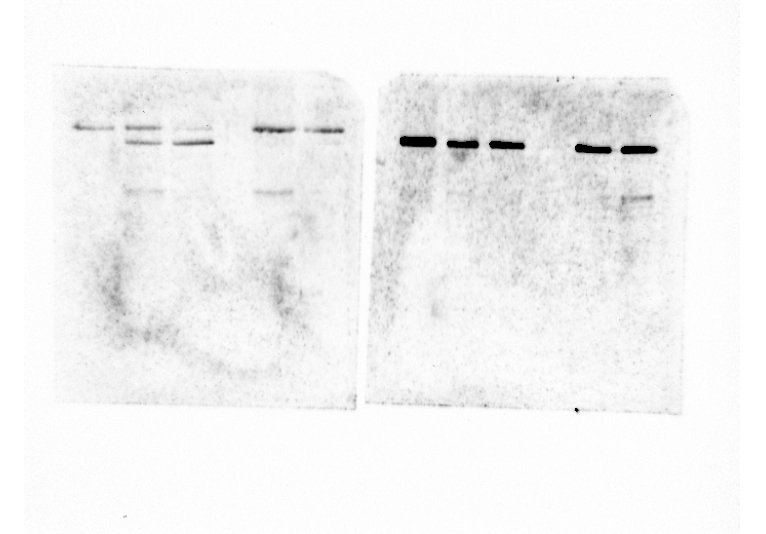

Supplement: Figure 2—source data 2. [file elife-73675-fig2-data2.zip › Figure 2-source data 2/WB_GSDMD-2.tif]

Figure 4 - source data 2

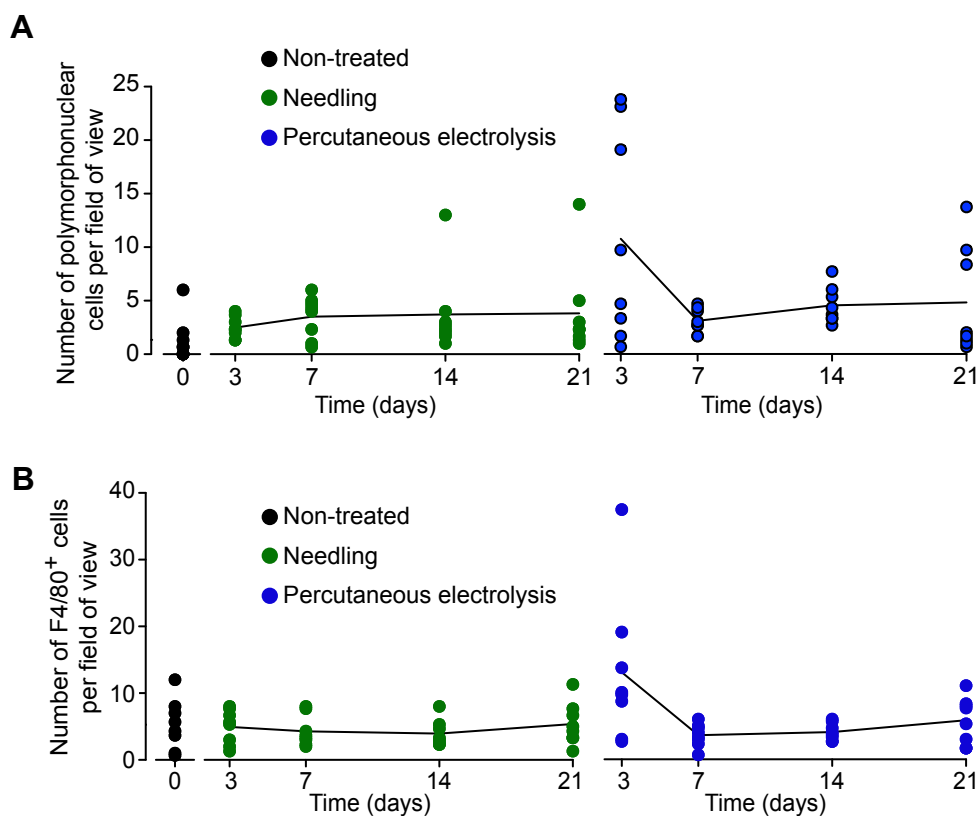

Supplement: Figure 4—source data 2. [file elife-73675-fig4-data2.pdf]
