# Supplementary material for: Galvanic current activates the NLRP3 inflammasome to promote Type I collagen production in tendon
Source: eLife. 2022 Feb 24;11:e73675. doi: 10.7554/eLife.73675 (PMC8896827; doi:10.7554/eLife.73675)
Supplement: Transparent reporting form [file elife-73675-transrepform1.pdf]

eLife's transparent reporting form

We encourage authors to provide detailed information *within their submission* to facilitate the interpretation and replication of experiments. Authors can upload supporting documentation to indicate the use of appropriate reporting guidelines for health-related research (see EQUATOR Network), life science research (see the BioSharing Information Resource), or the ARRIVE guidelines for reporting work involving animal research. Where applicable, authors should refer to any relevant reporting standards documents in this form.

If you have any questions, please consult our Journal Policies and/or contact us: editorial@elifesciences.org.

Sample-size estimation

- You should state whether an appropriate sample size was computed when the study was being designed
- You should state the statistical method of sample size computation and any required assumptions
- If no explicit power analysis was used, you should describe how you decided what sample (replicate) size (number) to use

Please outline where this information can be found within the submission (e.g., sections or figure legends), or explain why this information doesn't apply to your submission:

- Referring to the sample size used in figures 1, 2 and 3, we designed and performed the experiments based in our previous publications (Baroja-Mazo et al., 2014; Martín-Sánchez et al., 2016). This information can be found in figure legends, where each dot represents the result obtained in each experimental replicate of at least 3 independent experiments. Concerning figures 4, 5, 6 and 7, the animals sample size was determined based in a pilot study previously done by the group. This information can be found in figure legends, where each dot represents one independent mouse. The sample size was initially determined taking into account that we want to obtain a statistical power of 95% and a significant difference between the results of 0.05% using a restrictive nonparametric test.
- For the statistical analysis of two samples, we have used the nonparametric Mann-Whitney test when our samples are not normally distributed and parametric *t*-test when they are. For the statistical analysis of more than two samples, we have used nonparametric Kruskal-Wallis test when our samples are not normally distributed and parametric one-way ANOVA when they are. All the tests have been done assuming that our work is done in independent sample groups and we have independence of observations. This information can be found in the figure legends.
- For figures 1, 2 and 3, as the *in vitro* approach was exploratory at the beginning and the final results were unknown, we determined our sample size with $n \geq 3$ initial replicates, when a significant difference was found, then we used different experimental approaches to investigate the mechanisms that in some cases increases the n number of basic control experiments. For figures 4, 5, 6 and 7 we based our *in vivo* sample size accounting for the 3Rs principles: replacement, reduction and refinement. The sample size can be found in figure legends.

Replicates

- You should report how often each experiment was performed
- You should include a definition of biological versus technical replication

- The data obtained should be provided and sufficient information should be provided to indicate the number of independent biological and/or technical replicates
- If you encountered any outliers, you should describe how these were handled
- Criteria for exclusion/inclusion of data should be clearly stated
- High-throughput sequence data should be uploaded before submission, with a private link for reviewers provided (these are available from both GEO and ArrayExpress)

Please outline where this information can be found within the submission (e.g., sections or figure legends), or explain why this information doesn't apply to your submission:

- The experiments were performed in sets of animals that always included all different groups (different genetic background, control, needling and galvanic current) analyzed. The experiments were repeated to minimize variability and gain statistical power respecting the 3Rs principles. For *in vitro* studies, experiments were performed more or less every week.
- A biological replicate accounts for the results obtained from one independent single mouse generating only one data set (including the bone-marrow derived macrophages referred in figures 1, 2 and 3). A technical replicate accounts for the results obtained using a single mouse sample with the same treatment and generating two or more data sets.
- In this paper, we have used technical and biological replicates. The number of technical and biological replicates can be found in figure legends.
- Once we had all data and before performing the statistical analysis, to identify possible outliers we used the ROUT method (robust regression followed by outlier identification) with Prism (GraphPad). Q value was set to 1% in order to control the False Discovery Rate, meaning that less than 1% of the "statistically significant" findings will be false positives.
- See *table 1* below for additional criteria for exclusion/inclusion.
- Our study did not generated high-throughput sequence data. Do not apply.

Table 1

Figure	Explanation	Inclusion criteria	Exclusion criteria
1, 2 and 3	*In vitro* experiments from bone marrow-derived macrophages.	-	**Extracellular LDH value as measurement of cell viability**. When negative controls culture wells presented an elevated cell death.
4, 5, 6 and 7	*In vivo* experiments involving galvanic current procedure.	The animals underwent successful galvanic current stimulation.	The animals underwent non-successful galvanic current stimulation.

Statistical reporting

- Statistical analysis methods should be described and justified
- Raw data should be presented in figures whenever informative to do so (typically when N per group is less than 10)
- For each experiment, you should identify the statistical tests used, exact values of N, definitions of center, methods of multiple test correction, and dispersion and precision measures (e.g., mean, median, SD, SEM, confidence intervals; and, for the major substantive results, a measure of effect size (e.g., Pearson's r, Cohen's d)

- Report exact p-values wherever possible alongside the summary statistics and 95% confidence intervals. These should be reported for all key questions and not only when the p-value is less than 0.05.

Please outline where this information can be found within the submission (e.g., sections or figure legends), or explain why this information doesn't apply to your submission:

All the information accounting for statistical reporting is stated in material and method section, as well as in figure legends.
All the graphs represent raw data (each dot represents the value of an individual animal or *in vitro* experimental replicate) and p-value is represented in all matter questions.

(For large datasets, or papers with a very large number of statistical tests, you may upload a single table file with tests, Ns, etc., with reference to sections in the manuscript.)

Group allocation

- Indicate how samples were allocated into experimental groups (in the case of clinical studies, please specify allocation to treatment method); if randomization was used, please also state if restricted randomization was applied
- Indicate if masking was used during group allocation, data collection and/or data analysis

Please outline where this information can be found within the submission (e.g., sections or figure legends), or explain why this information doesn't apply to your submission:

Experimental group allocation does not apply to our submission, as all the experimental groups are from homogenous cell cultures wells plated form the same cell differentiation plates and the same day, or from matched age/sex animals. Therefore, the initial application of the different treatments is not conditioned to group allocation.

Additional data files ("source data")

- We encourage you to upload relevant additional data files, such as numerical data that are represented as a graph in a figure, or as a summary table
- Where provided, these should be in the most useful format, and they can be uploaded as "Source data" files linked to a main figure or table
- Include model definition files including the full list of parameters used
- Include code used for data analysis (e.g., R, MatLab)
- Avoid stating that data files are "available upon request"

Please indicate the figures or tables for which source data files have been provided:

All the graphs presented in the figures include raw data (each dot represents the value of an individual animal or *in vitro* experimental replicates).

eLife Sciences Publications, Ltd is a limited liability non-profit non-stock corporation incorporated in the State of Delaware, USA, with company number 5030732, and is registered in the UK with company number FC030576 and branch number BR015634 at the address 1st Floor, 24 Hills Road, Cambridge CB2 1JP | August 2014
